# Posttranscriptional regulation of 14‐3‐3*ζ* by RNA‐binding protein HuR modulating intestinal epithelial restitution after wounding

**DOI:** 10.14814/phy2.12858

**Published:** 2016-07-08

**Authors:** Natasha Z. Hansraj, Lan Xiao, Jing Wu, Gang Chen, Douglas J. Turner, Jian‐Ying Wang, Jaladanki N. Rao

**Affiliations:** ^1^ Cell Biology GroupDepartment of SurgeryUniversity of Maryland School of MedicineBaltimoreMaryland; ^2^ Baltimore Veterans Affairs Medical CenterBaltimoreMaryland; ^3^ Department of PathologyUniversity of Maryland School of MedicineBaltimoreMaryland

**Keywords:** Epithelial cell migration, mucosal injury, posttranscriptional regulation, rapid epithelial repair

## Abstract

The 14‐3‐3ζ is a member of the family of 14‐3‐3 proteins and participates in many aspects of cellular processes, but its regulation and involvement in gut mucosal homeostasis remain unknown. Here, we report that 14‐3‐3ζ expression is tightly regulated at the posttranscription level by RNA‐binding protein HuR and plays an important role in early intestinal epithelial restitution after wounding. The 14‐3‐3ζ was highly expressed in the mucosa of gastrointestinal tract and in cultured intestinal epithelial cells (IECs). The 3′ untranslated region (UTR) of the *14‐3‐3ζ* mRNA was bound to HuR, and this association enhanced 14‐3‐3ζ translation without effect on its mRNA content. Conditional target deletion of HuR in IECs decreased the level of 14‐3‐3ζ protein in the intestinal mucosa. Silencing 14‐3‐3ζ by transfection with specific siRNA targeting the *14‐3‐3ζ* mRNA suppressed intestinal epithelial restitution as indicated by a decrease in IEC migration after wounding, whereas ectopic overexpression of the wild‐type 14‐3‐3ζ promoted cell migration. These results indicate that HuR induces 14‐3‐3ζ translation via interaction with its 3′ UTR and that 14‐3‐3ζ is necessary for stimulation of IEC migration after wounding.

## Introduction

The mammalian gastrointestinal (GI) epithelium is among the most rapidly self‐renewing tissues in the body and it constitutes a dynamic physical barrier segregating the luminal content from the mucosal tissue (Silen and Ito 1985; Sato and Clevers 2013; Xiao et al. 2013). The integrity of GI epithelium depends on a balance among cell proliferation, migration, differentiation, and apoptosis (Wang and Johnson 1994; Gunther et al. 2013). Early epithelial restitution is an important repair modality in the mucosa and occurs as a consequence of epithelial cell migration over the damaged area after superficial injury, a process that is independent of cell proliferation (Rutten and Ito 1983; Nusrat et al. 1992; Dignass et al. 1994). Defective regulation of this process underlies various critical pathological conditions such as mucosal injury and bleeding, delayed healing, and epithelial barrier dysfunction (Hines et al. 2000; Guo et al. 2002, 2003; Yu et al. 2011). This rapid re‐epithelialization is a complex process that is regulated by numerous extracellular and intracellular factors, but its exact mechanism at cellular and molecular levels remains largely unknown.

In mammalian cells, regulation of the mRNA stability and translation is a critical step in the control of gene expression (Wang 2007; Wang et al. 2010; Zhuang et al. 2013; Liu et al. 2015). RNA‐binding proteins (RBPs) and microRNAs are two classes of *trans*‐acting factors that determine the posttranscriptional fate of mRNAs by interacting with their specific *cis*‐elements that are commonly located in 3′ untranslated regions (3′ UTRs) of many labile mRNAs (Keene 2007; Mendell and Olson 2012). The Hu‐antigen R (HuR) protein is the ubiquitously expressed member of the *ELAV*‐like family of RBPs and it has two N‐terminal RNA recognition motifs (RRMs), followed by a nucleocytoplasmic shuttling sequence, and a C‐terminal RRM (Abdelmohsen et al. 2007; Papadaki et al. 2009). HuR is predominantly located in the nucleus in unstimulated cells but it is rapidly translocated to the cytoplasm, where it directly interacts with and regulates mRNA stability and/or translation in response to specific stimuli (Antic et al. 1999; Hinman and Lou 2008; Young et al. 2009). We recently demonstrated that HuR regulates intestinal epithelial homeostasis by modulating intestinal epithelial cell (IEC) proliferation, migration, and apoptosis (Wang et al. 2010; Zhuang et al. 2013; Liu et al. 2015), whereas conditional deletion of HuR in IECs, intestinal epithelial cells (IECs) represses growth of the small intestinal mucosa by downregulating Wnt signaling (Liu et al. 2014).

The family of 14‐3‐3 proteins consists of seven members including *β*,* γ*,* ε*,* ζ*,* η*,* σ*, and *τ* in mammals, which are highly conserved and bind to the phosphoserine/threonine‐containing motifs (Sluchanko and Gusev 2010; Zhao et al. 2011). Among all seven members, the 14‐3‐3ζ protein is widely expressed in various tissues and is one of the most studied protein isoforms (Neal and Yu 2010; Chen et al. 2014). 14‐3‐3ζ regulates actin cytoskeleton remodeling, alters cellular adhesion, and affects cell migration via distinct cellular mechanisms (Louvet‐Vallée 2000; Pujuguet et al. 2003; Sluchanko and Gusev 2010; O'Toole et al. 2011). 14‐3‐3*ζ* directly interacts with and regulates ezrin expression and promotes cell migration by regulating the formation of membrane ruffles (Deakin et al. 2009; Chen et al. 2014). In human prostate cancer, silencing 14‐3‐3*ζ* inhibits Rac1 activation and decreases lamellipodia formation (Deakin et al. 2009; Sluchanko and Gusev 2010; Goc et al. 2012). 14‐3‐3*ζ* is highly expressed in gastric cancer cells and its content is clinically correlated with the size of the tumor (Hengstschläger et al. 2003; Jang et al. 2004). Here, we report that 14‐3‐3*ζ* expression is regulated at the posttranscription level by HuR and further reveal that 14‐3‐3ζ is necessary for stimulation of IEC migration during restitution after wounding.

## Materials and Methods

### Chemicals and cell culture

Tissue culture medium and heat‐inactivated fetal bovine serum (FBS) were from Invitrogen (Carlsbad, CA) and biochemicals were from Sigma (St. Louis, MO). The antibodies against HuR and 14‐3‐3ζ were from Santa Cruz Biotechnology (Santa Cruz, CA). The secondary antibody conjugated to horseradish peroxidase was purchased from Sigma‐Aldrich (St. Louis, MO).

The line of IEC‐6 cells (Quaroni et al. 1979) was purchased from the American Type Culture Collection (ATCC) (Manassas, VA) at passage 13. IEC‐6 cells were derived from normal rat intestinal crypt cells and were maintained in T‐150 flasks in Dulbecco's modified Eagle's medium (DMEM) supplemented with 5% heat‐inactivated FBS, 10 *μ*g/mL insulin, and 50 *μ*g/mL gentamicin sulfate. Flasks were incubated at 37°C in a humidified atmosphere of 90% air–10% CO_2_, and passages 16–20 were used in the experiments. Stable Cdx2‐transfected IEC‐6 (IEC‐Cdx2L1) cells were developed by Suh and Traber (Suh and Traber 1996) and maintained as described in our previous publications (Rao et al. 1999, 2002, 2008, 2010; Rao and Wang 2010; Rathor et al. 2014a,b). Caco‐2 cells were purchased from ATCC and maintained in standard culture conditions as described previously (Rao et al. 2008; Zou et al. 2016).

### Animal studies

All experiments were approved according to the animal experimental ethics committee guidelines by the University of Maryland Baltimore Institutional Animal Care and Use Committee. Mice were housed and handled in a specific pathogen‐free breeding barrier and cared by trained technicians and veterinarians. The strategy to generate and genotype IE‐HuR^−/−^ mice was described in our previous publication (Liu et al. 2014). The HuR‐floxed mouse has been described elsewhere (Katsanou et al. 2009), and HuR^flox/flox^ (HuR^fl/fl^) was crossed with Villin‐Cre mice (The Jackson Laboratory, Bar Harbor, ME) to yield IE‐HuR^−/−^ mice. HuR^fl/fl^‐Cre^−^ mice served as littermate control. Animals were euthanized by CO_2_ asphyxiation followed by cervical dislocation. A 4‐cm small intestinal segment taken from 0.5 cm distal to the ligament of Treitz and the segment of middle colon were collected. The mucosa was scraped from the underlying smooth muscle with a glass microscope slide and used for measurements of the levels of 14‐3‐3ζ and *HuR* mRNAs and proteins.

### RNA interference and plasmid construction

The small interfering (si)RNA that was designed to specifically target the coding region of HuR (siHuR) or *14‐3‐3ζ* (si14‐3‐3*ζ*) mRNA were purchased from Santa Cruz Biotechnology. Scrambled control siRNA (C‐siRNA), which had no sequence homology to any known genes, was used as the control. The siHuR, si14‐3‐3*ζ*, or C‐siRNA was transfected into cells as described previously (Rao et al. 2002, 2008, 2010; Chung et al. 2015). Briefly, for each 60‐mm cell culture dish, 20 *μ*L of the 5 *μ*mol/L stock siHuR, si14‐3‐3*ζ*, or C‐siRNA was mixed with 500 *μ*L of Opti‐MEM medium (Invitrogen). This mixture was added to a solution containing LipofectAMINE 2000 in 500 *μ*L of Opti‐MEM. The solution was incubated for 20 min at room temperature and gently overlaid onto monolayers of cells in 3 mL of medium, and cells were harvested for various assays after 48 h incubation.

The chimeric firefly luciferase reporter constructs containing different fragments of *14‐3‐3ζ* mRNA were generated by the procedure described previously (Wang et al. 2010; Liu et al. 2015). The full‐length 14‐3‐3*ζ* 5′ UTR, CR, or different fragments of 3′ UTR were amplified and subcloned into the pmirGLO‐Luciferase miRNA Target Expression Vector (Promega, Madison, WI) to generate the pmirGLO‐LUC‐14‐3‐3*ζ* – 5′ UTR, pmirGLO‐LUC‐14‐3‐3*ζ* – CR, and pmirGLO‐LUC‐14‐3‐3*ζ* – 3′ UTR (F1–F3). The sequence and orientation of the fragment in the luciferase reporter were confirmed by DNA sequencing and enzyme digestion.

### Western blot analysis

Whole‐cell lysates were prepared using 2% SDS, sonicated, and centrifuged (15,000 *g*) at 4°C for 15 min. The supernatants were boiled for 5 min and size fractionated by SDS‐PAGE (12% acrylamide). After transferring proteins onto nitrocellulose filters, the blots were incubated with primary antibodies recognizing 14‐3‐3*ζ* or HuR; following incubations with secondary antibodies, immunocomplexes were developed by chemiluminescence.

### RT‐PCR and real‐time quantitative PCR analysis

Total RNA was isolated by RNeasy mini kit (Qiagen, Valencia, CA) and used in reverse transcription and PCR amplification reactions as described previously (Rao et al. 2002, 2008; Zou et al. 2016). Real‐time quantitative PCR (q‐PCR) analysis was performed using 7500‐Fast Real‐Time PCR Systems with specific primers, probes, and software (Applied Biosystems, Foster City, CA).

### Biotin pull‐down assays and ribonucleoprotein immunoprecipitation analysis

The synthesis of biotinylated RNA was carried out as described previously (Wang et al. 2010; Zhuang et al. 2013; Zou et al. 2016). Since there are multiple predicted HuR binding sites in the 3′ UTR of the *14‐3‐3ζ* mRNA based on bioinformatic analysis, immunoprecipitation (IP) of ribonucleoprotein (RNP) assays was performed. Complementary DNA from IEC‐6 cells was used as a template for PCR amplification of 5′ UTR, CR, and 3′ UTR fragments of the *14‐3‐3ζ* mRNA. The 5′ primers contained the T7 RNA polymerase promoter sequence (T7, CCAAGCTTCTAATACGAC–TCACTATAGGGAGA). All sequences of oligonucleotides for the preparation of full‐length 5′ UTR, CR, and various short RNA probes for mapping the 14‐3‐3*ζ* 3′ UTR were described in Table 1. PCR‐amplified products were used as templates to transcribe biotinylated RNAs by T7 RNA polymerase in the presence of biotin–cytidine 5′ triphosphate as described previously (Wang et al. 2010; Zhuang et al. 2013). Biotinylated transcripts (6 *μ*g) were incubated with 120 *μ*g of cytoplasmic lysates for 30 min at room temperature. Complexes were isolated with paramagnetic streptavidin‐conjugated Dynabeads (Dynal, Oslo, Norway) and analyzed by western blot analysis.

**Table 1:** Oligonucleotide sequences of primers for 14‐3‐3ζ and Luc constructs

Name	Sequences
5′UTR	Forward 5′‐CCAAGCTTCTAATACGACTCACTATAGGGAGATTTCTCCTTCCCCTTCTTCC‐3′
	Reverse 5′‐CCAGCTCATTTTTATCCATGAC‐3′
CR	Forward 5′‐CCAAGCTTCTAATACGACTCACTATAGGGAGACAAACCTTGCTTCTAGGAGATAAAA‐3′
	Reverse 5′‐GTTGGAAGGCCGGTTAATTT‐3′
3′UTR‐F1	Forward 5′‐CCAAGCTTCTAATACGACTCACTATAGGGAGAAATTAACCGGCCTTCCAACT‐3′
	Reverse 5′‐GCAAAAATAATGAAAGCTAACAGC‐3′
3′UTR‐F2	Forward 5′‐CCAAGCTTCTAATACGACTCACTATAGGGAGATAAGGGCAGAAACGGTTCAC‐3′
	Reverse 5′‐GCCAAAATTTTAAATGGAACACA‐3′
3′UTR‐F3	Forward 5′‐CCAAGCTTCTAATACGACTCACTATAGGGAGATTTTGGCATATGGCATTTTCT‐3′
	Reverse 5′‐TTTCTCCATTGCACTTTTATTTGA‐3′
5′UTR‐Luc	Forward 5′‐TTTCTCCTTCCCCTTCTTCC‐3′
	Reverse 5′‐CCAGCTCATTTTTATCCATGAC‐3′
CR‐Luc	Forward 5′‐CAAACCTTGCTTCTAGGAGATAAAA‐3′
	Reverse 5′‐GTTGGAAGGCCGGTTAATTT‐3′
3′UTR‐F2‐Luc	Forward 5′‐TAAGGGCAGAAACGGTTCAC‐3′
	Reverse 5′‐GCCAAAATTTTAAATGGAACACA‐3′

To assess the association of endogenous HuR with endogenous *14‐3‐3ζ* mRNA, IP of RNP complexes was performed as described previously (Zhang et al. 2009; Wang et al. 2010; Liu et al. 2015; Zou et al. 2016). 20 million cells were collected per sample, and lysates were used for IP for 4 h at room temperature in the presence of excess (30 *μ*g) IP antibody (IgG, anti‐HuR). RNA in IP materials was used in RT followed by PCR and Q‐PCR analysis to detect the presence of 14‐3‐3*ζ* and *Gapdh* mRNA. *Stim1* mRNA was also examined and served as a positive control.

### Measurement of cell migration

Migration assays were carried out in three different lines of IECs as described previously (Rao et al. 1999, 2002, 2008, 2010; Rao and Wang 2010; Rathor et al. 2014a,b; Chung et al. 2015). Cells were plated at 6.25 × 10^4^/cm^2^ in Dulbecco's modified Eagle's medium containing FBS on 60‐mm dishes thinly coated with Matrigel following the manufacturer's instructions (BD Biosciences, San Diego, CA) and were incubated as described for stock cultures. Cells were fed on day 2, and cell migration was assayed on day 4. To initiate migration, cell layer was scratched with a single‐edge razor blade cut to ~27 mm in length. The scratch was made over the diameter of the dish and extended over an area 7‐ to 10‐mm wide. The migrating cells in six contiguous 0.1 mm^2^ were counted at ×100 magnification, beginning at the scratch line and extending as far out as the cells had migrated. All experiments were carried out in triplicate, and the results were reported as number of migrating cells per millimeter of scratch.

### Statistical analysis

All data are expressed as means ± SE from six dishes. Immunoblotting results were repeated three times. The significance of the difference between means was determined by analysis of variance. The level of significance was determined using the Duncan's multiple‐range test (Harter 1960).

## Results

### 14‐3‐3ζ protein is highly expressed in murine gut mucosa and cultured IECs

**Figure 1:**
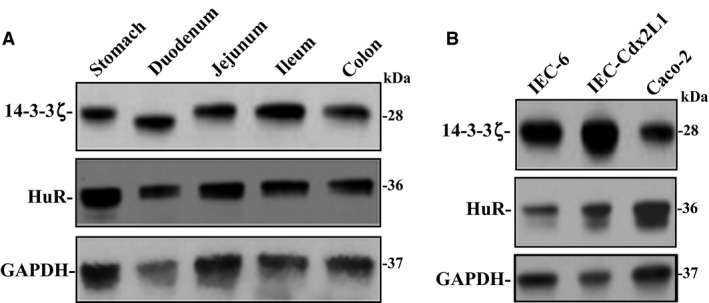
Levels of 14‐3‐3ζ and HuR in gut mucosa in mice and different lines of cultured IECs. (A) Representative immunoblots of 14‐3‐3ζ and HuR in the mucosa isolated from stomach, duodenum, jejunum, ileum, and colon as examined by western blot analysis. GAPDH immunoblotting was performed as an internal control for equal loading. (B) Representative immunoblots of 14‐3‐3ζ and HuR in IEC‐6, differentiated IEC‐Cdx2L1, and Caco‐2 cells. Three separate experiments were performed that showed similar results. IECs, intestinal epithelial cells.

First, we examined basal expression level of 14‐3‐3ζ protein in mucosal tissue obtained from different parts of GI tract. As shown in Figure 1A, both 14‐3‐3ζ and HuR proteins were highly expressed in the mucosal scrapings isolated from stomach, duodenum, jejunum, ileum, and colon as measured by western immunoblotting analysis. Second, we examined the 14‐3‐3ζ and HuR protein levels in three lines of cultured IECs including IEC‐6 cells, differentiated‐IEC‐Cdx2L1 cells, and Caco‐2 cells. IEC‐6 cells originated from intestinal crypts and are nontumorigenic and retain undifferentiated status of intestinal crypt cells (Quaroni et al. 1979), whereas IEC‐Cdx2L1 cells represent differentiated IECs (Suh and Traber 1996). Caco‐2 cells were derived from colon cancer cells. Consistent with observations in mucosal tissue, all three lines of IECs also expressed 14‐3‐3ζ and HuR proteins (Fig. 1B). These results indicate that 14‐3‐3ζ is highly enriched in the GI mucosa.

### HuR interacts with the 14‐3‐3ζ mRNA and regulates its translation

**Figure 2:**
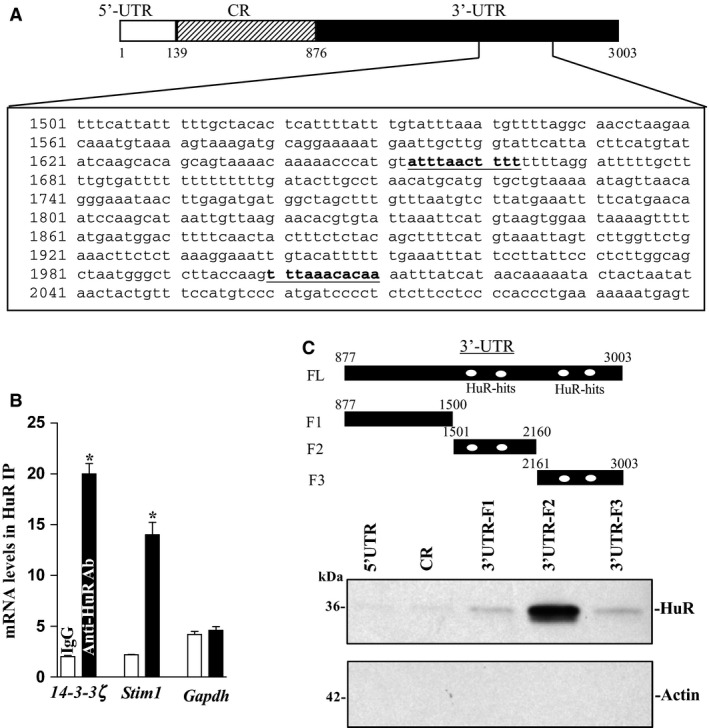
HuR binds the *14‐3‐3ζ* mRNA. (A) Schematic representation of the *14‐3‐3ζ* mRNA and the predicted hits of the HuR signature motif in its 3′ UTR. (B) Association of endogenous HuR with endogenous *14‐3‐3ζ* mRNA. After IP of RNA–protein complexes from cell lysates using either anti‐HuR antibody (Ab) or control IgG, RNA was isolated and used in reverse transcription reactions. Values were means ± SEM from triplicate samples. HuR binding to STIM1 was shown as a positive control; its binding to GAPDH served as a negative control in this study. (C) HuR binding to different fractions of 14‐3‐3ζ 5′ UTR, CR, and 3′ UTR. Top panel, schematic representation of the 14‐3‐3ζ 3′ UTR biotinylated transcripts. After incubation of cytoplasmic lysates with the full‐length (FL) or various fractions (F) of the 14‐3‐3ζ 3′ UTR, the resulting RNP complexes were pulled down, and the abundances of HuR and actin proteins in the pull‐down material were examined.

Given the predicted affinity of HuR for the 3′ UTR of the *14‐3‐3ζ* mRNA (Fig. 2A), we tested the hypothesis that HuR acts as a posttranscriptional regulator of 14‐3‐3ζ expression. First, we examined if there was an association between the *14‐3‐3ζ* mRNA and HuR in IECs by performing RNP IP assays using anti‐HuR antibody under conditions that preserved RNP integrity (Wang et al. 2010; Liu et al. 2015; Zou et al. 2016). This interaction was examined by isolating RNA from the IP material and subjecting it to reverse transcription (RT) followed by Q‐PCR analysis. 14‐3‐3*ζ* PCR products were highly enriched in HuR‐immunoprecipitated samples compared with control (IgG‐immunoprecipitated) samples (Fig. 2B), indicating that the *14‐3‐3ζ* mRNA is a target of HuR. The enrichment of STIM1 PCR product was also examined and served as a positive control (Fig. 2B), since HuR is shown to bind to the *Stim1* mRNA (Zhuang et al. 2013). The amplification of GAPDH PCR products, found in all samples as low‐level contaminating housekeeping transcripts (not HuR targets), served to monitor the evenness of sample input as reported previously (Wang et al. 2010; Liu et al. 2015; Zou et al. 2016).

Second, we tested whether HuR binds to 14‐3‐3*ζ* 5′ UTR, CR, or/and 3′ UTR by biotinylated transcripts which spanned different fractions of the *14‐3‐3ζ* mRNA as shown in Figure 2C (top panel). HuR directly interacted with the 14‐3‐3ζ 3′ UTR transcript, but it did not associate with the 14‐3‐3*ζ* 5′ UTR and CR transcripts as detected by western blot analysis of the pull‐down material (Fig. 2C, bottom panel). To further examine whether HuR binding to the 14‐3‐3ζ 3′ UTR is mediated through the specific sites containing predicted hits of the HuR motif, partial biotinylated transcripts spanning the 14‐3‐3ζ 3′ UTR were prepared (Fig. 2C, upper panel, schematic) and their associations with HuR were tested in pull‐down assays. HuR was found to specifically bind the F2 transcript (spanning positions 1501–2160), which contained HuR motif hits, without association with the F1 and F3 (although F3 also contained potential HuR hits). Together, these findings indicate that HuR interacts with the *14‐3‐3ζ* mRNA via its 3′ UTR rather than 5′ UTR and CR.

**Figure 3:**
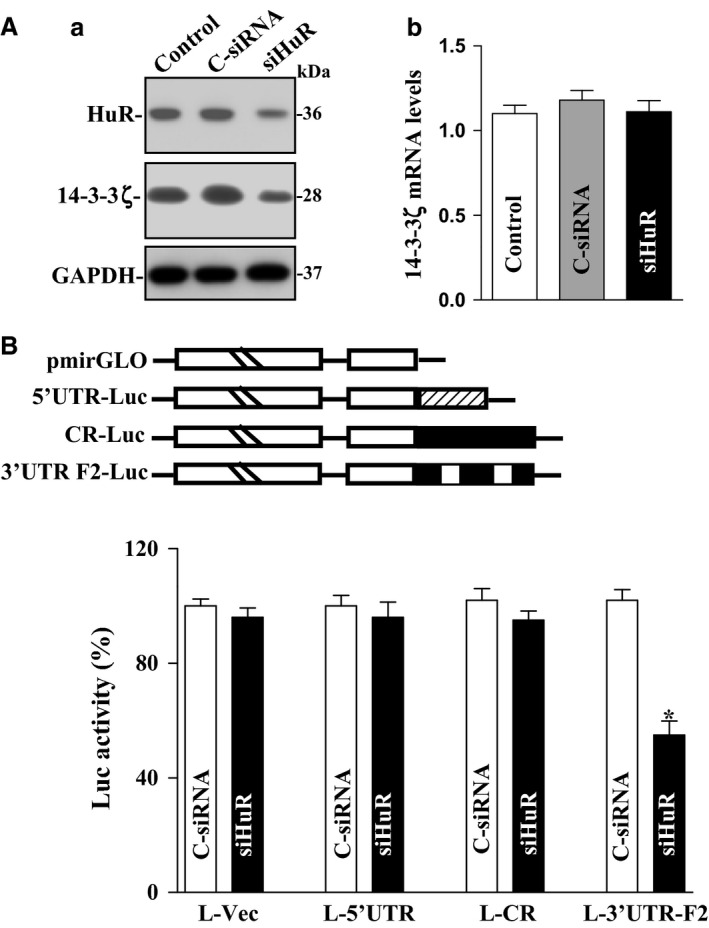
HuR silencing represses 14‐3‐3ζ translation in IECs. (Aa) Representative immunoblots of HuR and 14‐3‐3ζ. Cells were transfected with control siRNA (C‐siRNA) or siHuR, and whole‐cell lysates were harvested 48 h thereafter. Levels of HuR and 14‐3‐3ζ proteins were measured by western immunoblot analysis. (Ab) Levels of *14‐3‐3ζ* mRNA as measured by Q‐PCR analysis in cells treated as described in Aa. Values are the mean ± SEM (*n *=* *3). (B) Levels of reporter activities as measured by analysis of 14‐3‐3ζ 5′ UTR, CR, and 3′ UTR luciferase reporters in cells described in (Aa) Top panel, schematic of plasmids of different chimeric firefly luciferase 14‐3‐3ζ reporters. *P <* *0.05 compared with C‐siRNA. IECs, intestinal epithelial cells.

To directly examine the putative role of HuR in the 14‐3‐3ζ expression, siRNA targeting the *HuR* mRNA (siHuR) was used to reduce HuR levels. With >95% cells transfected (results not shown), siHuR silenced HuR expression, which was associated with a significant decrease in 14‐3‐3ζ protein levels (Fig. 3Aa). However, HuR silencing did not alter the levels of total *14‐3‐3ζ* mRNA (Fig. 3Ab). In order to determine the functional consequences of [*HuR/14‐3‐3ζ* mRNA] associations, we further examined the posttranscriptional activity by dual‐luciferase assays. We subcloned 14‐3‐3*ζ* 5′ UTR, CR, and 3′ UTR F2 into the pmirGLO dual‐luciferase miRNA target expression vector in order to generate pmirGLO – 5′ UTR, pmirGLO – CR, and pmirGLO – 3′ UTR F2 reporter constructs (Fig. 3B, schematic). HuR silencing by transfection with siHuR decreased the levels of 14‐3‐3*ζ* 3′ UTR F2 luciferase reporter activity but did not affect the activities of Luc 5′ UTR and Luc CR reporter genes (Fig. 3B, bottom panel). These results indicate that HuR interacts with and enhances 14‐3‐3*ζ* translation via its 3′ UTR.

### Tissue‐specific HuR deletion in IECs inhibits 14‐3‐3ζ expression in mice

**Figure 4:**
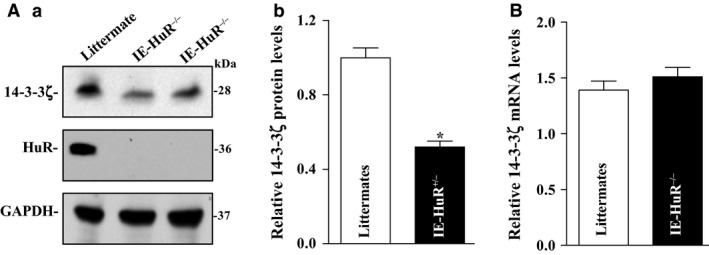
Conditional HuR deletion in IECs inhibits 14‐3‐3*ζ* expression. (Aa) Representative immunoblots of HuR and 14‐3‐3ζ in the small intestinal mucosa in IE‐HuR^−/−^ mice and control littermates. (Ab) Quantitative analysis of the immunoblotting signals as measured by densitometry. Values are the means ± SEM (*n *=* *5). *P <* *0.05 compared with IE‐control littermate mice. (B) Levels of *14‐3‐3ζ* mRNA in IE‐HuR^−/−^ mice as described in (A). IECs, intestinal epithelial cells.

To further investigate the role of HuR in the regulation of 14‐3‐3ζ expression, we examined changes in the levels of 14‐3‐3ζ protein in the small intestinal mucosa in IE‐HuR^−/−^ mice and control littermates. Consistent with our previous findings (Liu et al. 2014), *HuR* mRNA and protein were undetectable in the small intestinal and colonic mucosa in IE‐HuR^−/−^ mice, although HuR expression levels were normal in littermates. Specific deletion of HuR in IECs inhibited 14‐3‐3*ζ* expression, since the levels of 14‐3‐3*ζ* protein in the small intestinal mucosa decreased significantly in IE‐HuR^−/−^ mice compared to those observed in littermates (Fig. 4A). On the other hand, HuR deletion failed to alter total *14‐3‐3ζ* mRNA levels in the mucosa (Fig. 4B). These data indicate that the HuR positively regulates 14‐3‐3ζ translation in the intestinal mucosa.

### 14‐3‐3ζ is necessary for intestinal epithelial restitution after wounding

**Figure 5:**
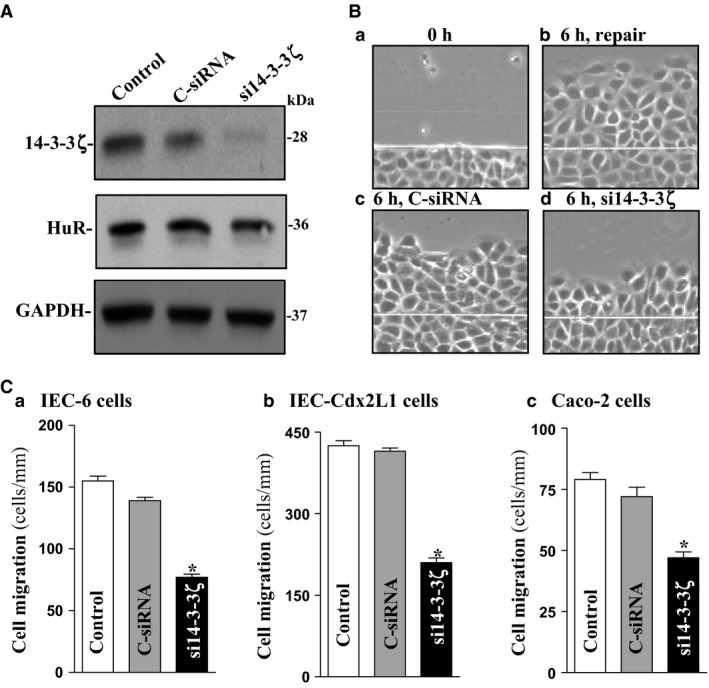
14‐3‐3ζ silencing inhibits cell migration after wounding in different lines of IECs. (A) Representative immunoblots of 14‐3‐3ζ and HuR. IEC‐6 cells were transfected with control siRNA (C‐siRNA) or si14‐3‐3ζ, and whole‐cell lysates were harvested 48 h thereafter. (B) Images of cell migration after wounding in IEC‐6 cells: (a) 0 h after wounding, (b) 6 h after wounding, (c) 6 h after wounding in cells transfected with C‐siRNA, and (d) 6 h after wounding in cells transfected with si14‐3‐3ζ. (C) Summarized data of cell migration 6 h after removal of part of the monolayer in (a) IEC‐6 cells, (b) IEC‐Cdx2L1 cells, and (c) Caco‐2 cells transfected with C‐siRNA or si14‐3‐3ζ for 48 h followed by cell migration assays. Data are means ± SEM from six dishes. *P <* *0.05 compared with cells transfected with C‐siRNA. IECs, intestinal epithelial cells.

To determine the involvement of 14‐3‐3ζ in the intestinal epithelial homeostasis, we examined its role in the regulation of intestinal epithelial restitution after wounding using an in vitro model that mimics proliferation‐independent rapid epithelial repair (Rao et al. 1999, 2002, 2008, 2010; Rao and Wang 2010; Rathor et al. 2014a; Chung et al. 2015). siRNA targeting *14‐3‐3ζ* mRNA (si14‐3‐3ζ) was used to specifically block endogenous 14‐3‐3ζ in IEC‐6 cells. As shown in Figure 5A, transfection of IEC‐6 cells with si14‐3‐3ζ for 48 h decreased 14‐3‐3ζ protein levels by ~90%, but it did not affect HuR content. 14‐3‐3ζ silencing impaired epithelial restitution after wounding (Fig. 5Ca), and the number of cells migrating over the denuded area 6 h after wounding was decreased by ~50% in 14‐3‐3ζ‐silenced cells. Neither 14‐3‐3ζ level nor cell migration was affected in cells transfected with control siRNA (C‐siRNA) at the same concentrations. We also examined the effect of 14‐3‐3ζ silencing on epithelial restitution in other cell lines and demonstrated that decreased levels of 14‐3‐3ζ by transfection with si14‐3‐3ζ also repressed cell migration after wounding in differentiated IEC‐Cdx2L1 cells and Caco‐2 cells (Fig. 5Cb,c). In addition, 14‐3‐3ζ silencing did not affect cell viability as measured by Trypan blue staining (data not shown).

**Figure 6:**
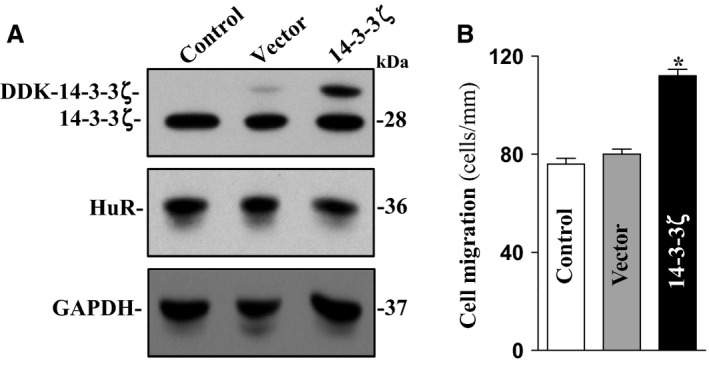
Ectopic overexpression of 14‐3‐3ζ enhances cell migration after wounding. (A) Representative immunoblots of 14‐3‐3ζ and HuR protein. Caco‐2 cells were transiently transfected with the 14‐3‐3ζ expression vector or empty vector (Null), and the levels of 14‐3‐3ζ and HuR were examined by western immunoblotting analysis 48 h after the transfection. (B) Summarized data showing cell migration 6 h after removal of part of the monolayer in cells described in A. Values are means ± SEM from six dishes. *P <* *0.05 compared with the cells transfected with empty vector.

To further define the role of 14‐3‐3ζ in epithelial restitution, we examined the effect of 14‐3‐3ζ overexpression on cell migration after wounding. Myc‐DDK tagged expression vector encoding the full‐length 14‐3‐3ζ cDNA under the control of the CMV promoter was used in this study, and it increased the level of DDK‐14‐3‐3ζ protein dramatically 48 h after the transfection. 14‐3‐3ζ overexpression enhanced cell migration after wounding (Fig. 6B). The numbers of cells migrating over the wounded edge in 14‐3‐3ζ‐transfected cells were increased by ~30% compared with those obtained from control cells or cells transfected with an empty vector when examined 6 h after wounding. These results strongly suggest that 14‐3‐3ζ plays an important role in the intestinal epithelial integrity by upregulating epithelial restitution after wounding.

## Discussion

The regulation of 14‐3‐3 family proteins have been extensively investigated (Neal and Yu 2010; Sluchanko and Gusev 2010; Obsilova et al. 2014), but a little is known about the distribution and biological function of 14‐3‐3ζ in the GI epithelium. The current study was highly focused on the role of 14‐3‐3ζ isoform in the regulation of rapid intestinal epithelial restitution, because this isoform is widely expressed in various tissues and it is involved in many aspects of cellular processes including cell motility (Louvet‐Vallée 2000; Pujuguet et al. 2003; Sluchanko and Gusev 2010; O'Toole et al. 2011). In the present study, we provide new evidence that 14‐3‐3ζ is highly expressed in the GI mucosa and its expression level is tightly regulated by HuR at the posttranscriptional level. Our findings also highlight a novel role of 14‐3‐3ζ in the regulation of intestinal epithelial restitution after wounding, thus advancing our understanding of the control of 14‐3‐3ζ expression and its cellular function in the gut epithelium.

The results reported here show that HuR bound to the *14‐3‐3ζ* mRNA by interacting with its 3′ UTR and affected 14‐3‐3ζ translation but did not alter total *14‐3‐3ζ* mRNA level. By various luciferase reporters bearing partial transcripts spanning the 14‐3‐3ζ 5′ UTR, CR, and 3′ UTR, our results further show that HuR associated with the 14‐3‐3ζ 3′ UTR F2 element (spanning positions 1501–2160) predominantly, but it failed to interact with the 5′ UTR or CR. Although we did not characterize the specific nucleotides with which HuR interacts and increases 14‐3‐3ζ translation, there are two predicted HuR‐hit motifs within the 14‐3‐3ζ 3′ UTR F2 region. The sequence spanning positions 2161–3003 (F3) of the 14‐3‐3ζ 3′ UTR also contains the computationally predicted HuR hits, but it did bind to HuR, suggesting that the sequences are inaccessible to HuR. It is possible that this potential HuR site is targeted by other RBPs which have greater binding affinity than HuR. These findings are consistent with observations in other studies showing that HuR binds to AREs commonly located at the 3′ UTRs of labile mRNAs, thus enhancing their stability and/or translation (Zhang et al. 2009; Wang et al. 2010; Zhuang et al. 2013; Liu et al. 2015). HuR silencing markedly reduced 14‐3‐3ζ protein levels but did not alter its mRNA level, suggesting that HuR regulates 14‐3‐3ζ translation rather than its mRNA stability.

Although the exact mechanism whereby HuR enhances *14‐3‐3ζ* is still unknown, several studies suggest that HuR acts by protecting the body of the mRNAs from degradation without slowing the rate of deadenylation (Slevin et al. 2007; Kim and Gorospe 2008). Previous studies revealed that 14‐3‐3 proteins associate with several serine‐phosphorylated RBPs such as AUF1, BRF1, and KSRP and influenced their localization and/or ability to bind specific mRNAs (Gherzi et al. 2004; Keene 2007). Kim et al. reported that 14‐3‐3 proteins participate in the retention of phosphorylated HuR in the nucleus (Kim et al. 2008), although there are no results showing the role of HuR in the regulation of expression of 14‐3‐3 proteins. Ongoing experiments are testing if the stimulatory effect of HuR on the *14‐3‐3ζ* translation depends on HuR phosphorylation (Kim and Gorospe 2008; Yu et al. 2011).

The 14‐3‐3*ζ* protein has biological function and plays an important role in the intestinal epithelial homeostasis. Expression of 14‐3‐3ζ is necessary for epithelial restitution after wounding, whereas 14‐3‐3ζ silencing repressed cell migration over the wounded area (Fig. 5). Consistent with our current findings, 14‐3‐3ζ is shown to regulate endothelial cell motility by modulating F‐actin cytoskeletal remodeling (Sluchanko and Gusev 2010; Toyo‐oka et al. 2014); the loss of 14‐3‐3ζ results in the stabilization of δ‐catenin, decreases *β*‐catenin expression, and thereby inactivates Rho family GTPases (Bialkowska et al. 2003). Absence of 14‐3‐3ζ was also found to inhibit integrin‐induced Rac1 activation and cell spreading in HeLa cells (Kobayashi et al. 2011; Goc et al. 2012). Depletion of 14‐3‐3ζ levels induces the endoplasmic reticulum (ER) stress and cell death in mouse hippocampal cultures (Heverin et al. 2012), and transgenic overexpression of 14‐3‐3ζ protects against ER stress and prevents neuronal cell death (Murphy et al. 2008; Brennan et al. 2013). Moreover, 14‐3‐3ζ interacts with ezrin and regulates cell motility by regulating formation of the membrane ruffles in HEK293 cells (Chen et al. 2014).

In summary, these results indicate that HuR regulates 14‐3‐3ζ expression through a direct interaction with the *14‐3‐3ζ* mRNA. HuR silencing in vitro or conditional HuR deletion in vivo decreases 14‐3‐3ζ protein levels but does not alter total *14‐3‐3ζ* mRNA content, suggesting the HuR modulates 14‐3‐3ζ translation rather than its mRNA stability. Expression of 14‐3‐3ζ is necessary for intestinal epithelial restitution after wounding, whereas 14‐3‐3ζ silencing represses cell migration over the wounded area. Since basal level of 14‐3‐3ζ is relatively high in the GI epithelium, regulation of 14‐3‐3ζ expression by HuR is physiologically significant in the intestinal epithelium integrity after injury.

## Conflict of Interest

None declared.
